# Friedewald, Martin/Hopkins ou Sampson/NIH: Qual o Melhor Método para Estimar o LDL-Colesterol?

**DOI:** 10.36660/abc.20220455

**Published:** 2022-07-29

**Authors:** Fernando Cesena

**Affiliations:** 1 Cenocor Guarulhos SP Brasil Cenocor, Guarulhos, SP – Brasil

**Keywords:** LDL-Colesterol, Hipercolesterolemia, Testes Laboratoriais/métodos, Hiperlipidemias

A doença cardiovascular aterosclerótica (DCVA) é a principal causa de morte no Brasil e na maior parte do mundo.^1^ A redução do colesterol da lipoproteína de baixa densidade (LDL-c) é o primeiro objetivo lipídico para prevenir a DCVA. A decisão sobre o início ou intensificação da terapia medicamentosa redutora de LDL-c é baseada no risco de eventos e no nível de LDL-c,^2^ portanto, uma determinação precisa do LDL-c é altamente desejável.

O método padrão-ouro para determinar a concentração plasmática de LDL-c é a β-quantificação, um procedimento caro e demorado baseado em ultracentrifugação e precipitação. Os métodos diretos que usam produtos químicos patenteados em vez de ultracentrifugação também são demorados e caros. Além disso, carecem de padronização e a precisão nem sempre é boa.^3^

Durante várias décadas, o LDL-c foi estimado por uma fórmula proposta por Friedewald na década de 1970.^4^ O LDL-c é dado subtraindo o HDL-colesterol (HDL-c) e o VLDL-colesterol (VLDL-c) do colesterol total, e o VLDL-c é estimado pela divisão dos triglicerídeos (TG) por um fator fixo de 5. O problema é que a fração de TG que estima o VLDL-c não é constante. Quando o nível de TG é alto, ou a concentração de LDL-c é baixa, a fórmula de Friedewald superestima o VLDL-c e, consequentemente, subestima o LDL-c. Quando o nível de TG é ≥400 mg/dL, a precisão da fórmula de Friedewald é inaceitavelmente baixa.^3^ A subestimação do LDL-c pode impedir o tratamento adequado e exacerbar o baixo alcance das metas de LDL-c, questão relevante no combate à DCVA.^5,6^

Outros métodos para calcular o LDL-c com mais precisão foram propostos, e o mais bem-sucedido até agora é a fórmula de Martin/Hopkins. Este método estima o VLDL-c dividindo os TG por um fator ajustável de acordo com os níveis de TG e do colesterol não-HDL.^7^ Essa equação é especialmente indicada quando o LDL-c é <70 mg/dL, os TG estão entre 175 e 400 mg/dL, ou em condições sem jejum, quando a fórmula de Friedewald tem mais limitações.^3,8^

Em 2020, outra equação foi proposta por Sampson et al. usando amostras do *National Institutes of Health*. O VLDL-c foi estimado por regressão múltipla pelo método dos mínimos quadrados. Os autores afirmam que a fórmula tem acurácia semelhante ou superior a outras abordagens e é útil para o cálculo do LDL-c em condições de níveis elevados de TG até 800 mg/dL.^9^

Nesse contexto, Naser et al.,^10^ relatam nos Arquivos Brasileiros de Cardiologia um estudo comparando o LDL-c calculado pelos métodos citados com o LDL-c medido diretamente em 402 pacientes com diabetes mellitus. Eles concluem que as equações de Martin/Hopkins e Sampson/NIH têm uma concordância semelhante com o LDL-c medido, um pouco melhor do que o observado com a fórmula de Friedewald. No entanto, todas as equações apresentaram desempenho ruim quando a concentração de TG foi >400 mg/dL.^10^ Embora o comparador utilizado neste estudo (método direto) seja passível de críticas, como apontado acima, o trabalho contribui para o conhecimento ao avaliar a acurácia relativa do método Sampson/NIH. Nesse sentido, evidências de dois grandes bancos de dados usando LDL-c medido diretamente como comparador favoreceram a abordagem de Martin/Hopkins,^11,12^ enquanto um estudo menor mostrou que a fórmula de Sampson/NIH teve maior concordância com o LDL-c estimado usando o VLDL-c medido por ultracentrifugação em indivíduos com hiperlipidemia familiar combinada.^13^

Dois estudos recentes publicados por Sajja et al.,^14,15^ da Universidade Johns Hopkins levantaram preocupações sobre o método Sampson/NIH. Em um deles, os autores mostraram que uma versão estendida da equação de Martin/Hopkins teve melhor precisão do que as fórmulas de Friedewald e Sampson/NIH em indivíduos com níveis de TG de 400 a 799 mg/dL. No entanto, a subestimação do LDL-c foi comum em níveis baixos com todos os métodos, especialmente Friedewald e Sampson/NIH. É importante ressaltar que a ausência de jejum não alterou o desempenho do método Martin/Hopkins, mas reduziu a precisão da fórmula de Sampson/NIH.^14^ Em outro trabalho, os autores demonstraram diferenças clinicamente significativas no LDL-c calculado por diferentes fórmulas em pacientes com DCVA. O LDL-c foi geralmente maior com a equação de Martin/Hopkins, sugerindo uma maior taxa de subestimação do LDL-c com os métodos de Friedewald e Sampson/NIH.^15^

Quais poderiam ser as recomendações práticas sobre a estimativa de LDL-c? Em níveis baixos de LDL-c ou altos de TG, o clínico deve lembrar que o LDL-c calculado pode estar subestimado, principalmente se a fórmula de Friedewald foi usada. Assim, as diretrizes atuais recomendam o método Martin/Hopkins quando o LDL-c é <70 mg/dL ou o nível de TG é 175-400 mg/dL.^3^ Embora a nova equação Sampson/NIH tenha se mostrado consistentemente melhor do que a fórmula de Friedewald, a precisão comparável à do método Martin/Hopkins tem sido questionada, e seu uso rotineiro deve esperar por mais dados de validação.

Quando o nível de TG é >400 mg/dL, o LDL-c é melhor determinado pelo método direto. A medição da apolipoproteína B e o cálculo do nível de colesterol não-HDL também são úteis para refinar a estratificação de risco e ajudar nas decisões clínicas.^3^

Estimar o LDL-c por equações é uma questão em evolução. Os métodos mais recentes superaram a antiga fórmula de Friedewald. Em níveis baixos de LDL-c ou altas concentrações de TG (especialmente ≥400 mg/dL), recomenda-se cautela no cálculo de LDL-c devido à chance de subestimação e subtratamento.
