# Supplementary material for: Friedewald, Martin/Hopkins ou Sampson/NIH: Qual o Melhor Método para Estimar o LDL-Colesterol?
Source: Arq Bras Cardiol. 2022 Jul 29;119(2):234–5. [Article in Portuguese] doi: 10.36660/abc.20220455 (PMC9363056; doi:10.36660/abc.20220455)
Supplement: Translation

# Translation: Friedewald, Martin/Hopkins, or Sampson/NIH: Which is the Best Method to Estimate LDL-Cholesterol?

**Authors:** Fernando Cesena

**Affiliations:** 1 Guarulhos SP Brazil Cenocor, Guarulhos, SP – Brazil

**Keywords:** Cholesterol-LDL, Hypercholesterolemia, Laboratory Tests/methods, Hyperlipidemias

Atherosclerotic cardiovascular disease (ASCVD) is the main cause of death in Brazil and most of the world.^1^ Reduction in low-density lipoprotein cholesterol (LDL-c) is the first lipid goal to prevent ASCVD. The decision on the initiation or intensification of LDL-c lowering drug therapy is based on the risk of events and the LDL-c level,^2^ so an accurate determination of LDL-c is highly desirable.

The gold standard method to determine the plasma concentration of LDL-c is β-quantification, an expensive, time-consuming procedure based on ultracentrifugation and precipitation. Direct methods that use proprietary chemicals instead of ultracentrifugation are also time-consuming and costly. Moreover, they lack standardization, and the accuracy is not always good.^3^

For several decades, LDL-c has been estimated by a formula proposed by Friedewald in the 1970s.^4^ LDL-c is given by subtracting HDL-cholesterol (HDL-c) and VLDL-cholesterol (VLDL-c) from total cholesterol, and VLDL-c is estimated by dividing triglycerides (TG) by a fixed factor of 5. The problem is that the fraction of TG that estimates VLDL-c is not constant. When the TG level is high, or the LDL-c concentration is low, the Friedewald formula overestimates VLDL-c and, consequently, underestimates LDL-c. When the TG level is ≥400 mg/dL, the accuracy of the Friedewald formula is unacceptably low.^3^ LDL-c underestimation may prevent appropriate treatment and exacerbate low achievement of LDL-c targets, a relevant issue in the fight against ASCVD.^5,6^

Other methods to calculate LDL-c more accurately have been proposed, and the most successful so far is the Martin/Hopkins formula. This method estimates VLDL-c by dividing TG by an adjustable factor according to TG and non-HDL-c levels.^7^ This equation is especially indicated when the LDL-c is <70 mg/dL, the TG are between 175 and 400 mg/dL, or in nonfasting conditions, when the Friedewald formula has more limitations.^3,8^

In 2020, another equation was proposed by Sampson et al. using samples from the National Institutes of Health. VLDL-c was estimated by multiple least squares regression. The authors state that the method has similar or greater accuracy than other approaches and is useful for calculating LDL-c in conditions of high TG levels up to 800 mg/dL.^9^

In this context, Naser et al.^10^ report in the *Arquivos Brasileiros de Cardiologia* a study comparing LDL-c calculated by the aforementioned methods with LDL-c directly measured in 402 patients with diabetes mellitus. They conclude that the Martin/Hopkins and Sampson/NIH equations have a similar agreement with measured LDL-c, slightly better than that observed with the Friedewald formula. However, all the equations showed poor performance when the TG concentration was >400 mg/dL.^10^ Although the comparator used in this study (direct method) is subject to criticism, as pointed out above, the work contributes to the knowledge while assessing the relative accuracy of the Sampson/NIH method. In this sense, evidence from two large databases using directly measured LDL-c as the comparator favored the Martin/Hopkins approach,^11,12^ whereas a smaller study found that the Sampson/NIH formula had higher concordance with LDL-c estimated using VLDL-c measured by ultracentrifugation in individuals with familial combined hyperlipidemia.^13^

Two recent studies published by Sajja et al.^14,15^ from Johns Hopkins University have raised concerns about the Sampson/NIH method. In one of them, the authors showed that an extended version of the Martin/Hopkins equation had better accuracy than the Friedewald and Sampson/NIH formulas in individuals with TG levels of 400 to 799 mg/dL. However, LDL-c underestimation was common at low levels with all the methods, especially Friedewald and Sampson/NIH. Importantly, a nonfasting state did not change the performance of the Martin/Hopkins method but reduced the accuracy of the Sampson/NIH formula.^14^ In another work, the authors demonstrated clinically meaningful differences in LDL-c calculated by different formulas in patients with ASCVD. LDL-c was usually higher with the Martin/Hopkins equation, suggesting a higher rate of LDL-c underestimation with the Friedewald and Sampson/NIH methods.^15^

What could be practical recommendations regarding LDL-c estimation? At low LDL-c levels or high TG concentrations, the clinician should remember that the calculated LDL-c may be underestimated, particularly if the Friedewald formula was used. Accordingly, current guidelines have recommended the Martin/Hopkins method when the LDL-c is <70 mg/dL or the TG level is 175-400 mg/dL.^3^ While the newer Sampson/NIH equation has performed consistently better than the Friedewald formula, accuracy comparable to that of the Martin/Hopkins method has been questioned, and its routine use should wait for more validation data.

When the TG level is >400 mg/dL, LDL-c is better determined by a direct method. Measurement of apolipoprotein B and calculation of non-HDL-c level are also useful to refine risk stratification and help clinical decisions.^3^

Estimating LDL-c by equations is an evolving issue. Newer methods have surpassed the old Friedewald formula. At low LDL-c levels or high TG concentrations (especially ≥400 mg/dL), caution is advised in calculating LDL-c due to the chance of underestimation and undertreatment.
